# The percentage of resected and ischemic volume determined by a geometric model is a significant predictor of renal functional change after partial nephrectomy

**DOI:** 10.1590/S1677-5538.IBJU.2015.0423

**Published:** 2017

**Authors:** Wei-Hsuan Huang, Chao-Hsiang Chang, Chi-Ping Huang, Hsi-Chin Wu, Po-Fan Hsieh

**Affiliations:** 1 Department of Urology, China Medical University Hospital, Taichung, Taiwan;; 2 School of Medicine, China Medical University, Taichung, Taiwan;; 3 Department of Urology, An-Nan Hospital, Tainan, Taiwan

**Keywords:** Nephrectomy, Delayed Graft Function, Kidney Neoplasms

## Abstract

**Purpose:**

The percentage of parenchyma preserved plays a predominant role in predicting renal function after partial nephrectomy (PN). Currently there is no standard method to estimate preserved renal parenchyma. In this study we propose a formula of the percentage of resected and ischemic volume (PRAIV) determined by a geometric model and evaluate the relationships between renal functional change and PRAIV as well as other clinical parameters.

**Materials and Methods:**

We identified 71 patients who underwent open PN between January 2004 and April 2014. Assuming the kidney to be an ellipsoid with bilaterally equal volume and tumor to be a sphere, we calculated PRAIV by integral calculus. Nadir estimated glomerular filtration rate (eGFR) between postoperative 3 and 12 months were recorded. The correlation between percent eGFR reduction, PRAIV, and other clinical parameters were examined.

**Results:**

On univariate analysis, age (p=0.03), depth of tumor invasion (p=0.004), C index (p=0.003), RAIV (p=0.04), and PRAIV (p<0.001) were correlated with percent reduction of eGFR. However, only age (p=0.007) and PRAIV (p<0.001) were significantly correlated with percent reduction of eGFR on multivariate analysis. Depicting these values along the regression line, we found R^2^ was 0.194 and 0.073 for PRAIV and age, respectively.

**Conclusions:**

PRAIV determined by a geometric model is a significant predictor of renal functional change after PN. Using PRAIV, we can estimate percent eGFR reduction preoperatively for better patient consultation and surgical planning.

## INTRODUCTION

Partial nephrectomy (PN) is currently the standard treatment of T1 renal tumors (1-3). Compared with radical nephrectomy, PN provides equivalent oncological control and better preservation of renal function (2). Multiple tumor factors (tumor size and complexity), patient factors (preoperative renal function, presence of a solitary kidney, age, sex, comorbidities), and surgical factors (ischemia type, ischemia duration, amount of preserved renal parenchyma) have been postulated to be associated with renal function after PN (4). Nephrometry systems including C-index, PADUA and RENAL scores were also found to have correlation with surgical complexity and change in renal function (5, 6). In studies which included the amount of preserved renal parenchyma to access postoperative renal function, the percentage of parenchyma preserved plays a predominant role in predicting renal function (7-9).

Several methods, such as intraoperative visual estimation and analysis of computerized tomography (CT) images, were proposed to estimate the amount of preserved renal parenchyma (7-13). Recently Shin et al. reported a formula using integral calculus to calculate the resected and ischemic volume (RAIV) during PN (14). In their study, RAIV had superior correlation with the absolute and percent change in estimated glomerular filtration rate (eGFR) compared to nephrometry systems including RENAL, PADUA, and C-index. However, the concept of percentage of parenchyma preserved was not included in RAIV. In other words, the same RAIV may cause different changes in patients with various renal parenchymal volumes. In this study we propose a new formula of percentage of RAIV (PRAIV) based on a geometric model. We also compare PRAIV with RAIV, nephrometry systems, and other clinical parameters in predicting the percent reduction of postoperative renal function.

## MATERIALS AND METHODS

Under the approval of institutional review board, we identified 71 patients who underwent open PN in a tertiary referral center between January 2004 and April 2014. We retrospectively analyzed their medical records and preoperative abdominopelvic CT or magnetic resonance imaging. Eight patients were excluded for incomplete recording of perioperative parameters. The principal techniques of PN included clamping of hilar vessels until completion of cortex sutures, commence of resection immediately after ice slush applying, and intravenous administration of mannitol as a reno-protective agent.

The cohort of 63 patients had bilateral kidneys. Serum creatinine was measured at a single clinical reference laboratory. Renal function was assessed by estimated glomerular filtration rate (eGFR) using the MDRD2 (Modification of Diet in Renal Disease 2) equation (15). Measurements of renal function were done immediately before operation, and nadir eGFR was recorded between postoperative 3 and 12 months.

**Figure 1:**
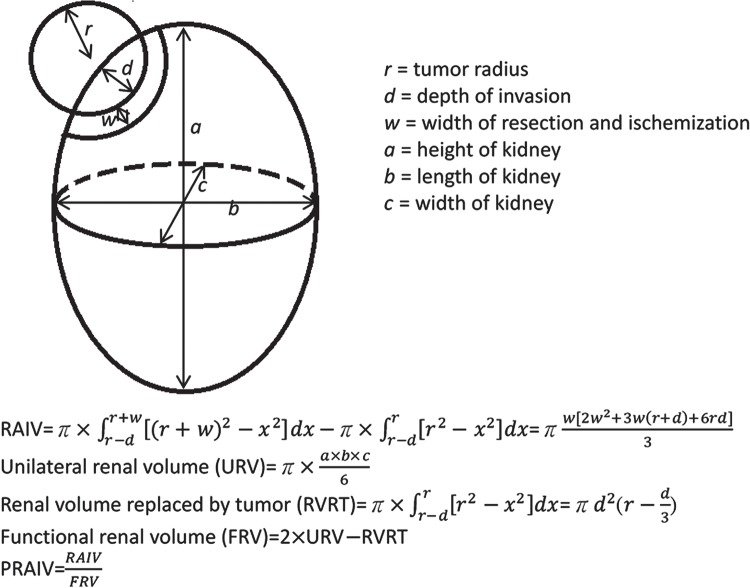
Illustration of geometric renal tumor model and calculation process of RAIV and PRAIV.

In addition to renal function, preoperative demographic information (age, gender, tumor size, depth of invasion) and perioperative parameters (cold ischemia time, estimated blood loss, pathologic report, RENAL, PADUA, C-index, RAIV, PRAIV) were recorded. RAIV was determined by the equation proposed by Shin et al. (14). Assuming the kidney to be an ellipsoid with bilaterally equal volume and tumor to be a sphere, we calculated PRAIV by dividing RAIV with functional renal volume (Figure-1).

### Statistical analysis

Univariate and multivariate analysis were done to access the relationship between percent reduction of eGFR and demographic and perioperative parameters. The relationships were plotted using simple regression. The coefficient of determination (R^2^) indicates how well a regression line fits data. Data analysis was done using the Statistical Package for Social Sciences (SPSS Inc., Chicago, IL, USA), version 17.0 for Windows with the null hypothesis rejected at p<0.05.

## RESULTS

Table 1 lists demographic and perioperative data of the study cohort. Mean age was 57.4 years, and 66.7% of the patients were male. Mean tumor diameter was 3.4cm, and mean depth of tumor invasion was 1.7cm. Renal cell carcinoma was diagnosed in 84.1% of the renal tumors. For the purpose of simplifying calculation of RAIV and PRAIV, the width of peritumor parenchymal resection and ischemization was empirically defined as 0.5 centimeter. Mean RAIV was 12.3cm3, and mean PRAIV was 4.4%. Mean RENAL, PADUA, and C-index were 6.9, 8.2, and 2.4, respectively. As for functional outcome, mean preoperative eGFR was 80mL/min/1.73m^2^, and eGFR reduced by a mean of 13.7% postoperatively.

**Table 1:** Demographic and perioperative data of 63 patients.

Gender		
No. male (%)	42	(66.7)
No. female (%)	21	(33.3)
Mean age (range)	57.4	(25-83)
Mean cm tumor diameter (range)	3.4	(1-15)
Mean cm depth of tumor invasion (range)	1.7	(0.1-3)
**No. pathology results**		
pT1a	48	
pT1b	5	
Benign	10	
Mean min cold ischemia time (range)	40.6	(6.6-71)
Mean mL blood loss (range)	330	(50-2100)
Mean RENAL (range)	6.9	(4-12)
Mean PADUA (range)	8.2	(6-13)
Mean C-index (range)	2.4	(0.8-6.7)
Mean cm3 RAIV (range)	12.3	(1.8-45.1)
Mean PRAIV (range)	4.4	(1-16)
**Mean mL/min/1.73m** ^**2**^ **eGFR (range)**		
Preop	80	(17-137)
Postop nadir	69.3	(12-115)
Mean % eGFR reduction	13.7	(-15-59)

**Figure 2:**
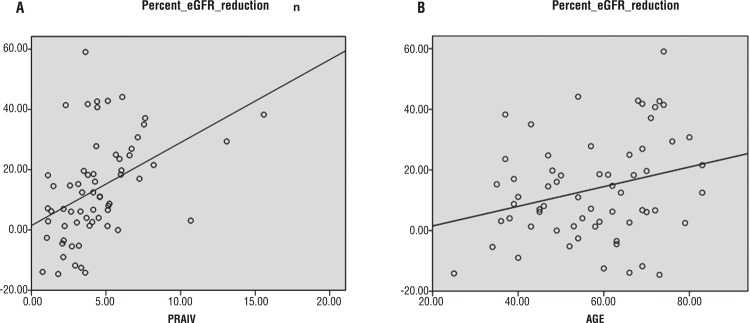
Correlation plots of percent eGFR reduction with PRAIV and age. A, percent eGFR reduction vs. PRAIV, y=1.512 + 2.752x, R2=0.194. B, percent eGFR reduction vs age, y=-4.958 + 0.324x, R2=0.073.

Table-2 shows the correlation between percent reduction of eGFR and clinical parameters. On univariate analysis, age (p=0.03), depth of tumor invasion (p=0.004), C-index (p=0.003), RAIV (p=0.04), and PRAIV (p<0.001) were correlated with percent reduction of eGFR. However, only age (p=0.007) and PRAIV (p<0.001) remained significantly correlated with percent reduction of eGFR on multivariate analysis. Depicting these values along the regression line, we found R^2^ was 0.194 and 0.073 for PRAIV and age, respectively (Figure-2). On the other hand, gender, tumor size, cold ischemia time, and preoperative renal function were not significantly associated with percent reduction of eGFR, while RENAL and PADUA had only marginal correlations with renal function change (p=0.05 and 0.07 on univariate analysis).

**Table 2:** Univariate and multivariate analysis of factors predicting percent eGFR reduction.

	Univariate		Multivariate	

	Beta	p value	Beta	p value
Gender	0.24	0.06	0.12	0.29
Age	0.27	0.03	0.31	0.007
Tumor size	0.07	0.6	-0.25	0.08
Depth of invasion	0.34	0.004	0.23	0.13
Cold ischemia time	0.12	0.36	0.18	0.1
Preop eGFR	-0.06	0.66	0.11	0.37
RENAL	0.25	0.05	0.13	0.27
PADUA	0.23	0.07	0.13	0.32
C-index	-0.37	0.003	-0.2	0.12
RAIV	0.26	0.04	-0.15	0.5
PRAIV	0.44	<0.001	0.47	<0.001

**Beta=** standardized coefficient

## DISCUSSION

Factors predicting functional change after PN have been an interesting field with many investigations. A strong correlation was found between the quality and quantity of renal parenchyma preserved and long-term renal function (16). Compared with ischemia duration, preoperative renal function, and other perioperative parameters, the percentage of renal parenchyma preserved had an even greater impact on ultimate renal function after PN (7-9). It was reported that a 5% increase in the amount of renal volume preserved carried a 17% reduction of the risk of stage 4 chronic kidney disease (17). In this study we investigated the influence of PRAIV on the functional outcome in a cohort of patients undergoing open PN. We found that PRAIV was the most important predictor of renal functional change.

Various methods in estimating resected renal volume were reported in literature. Theoretically the segmentation algorithm should be the most accurate. However, measuring areas on each axial section of CT scan was time-consuming and required sophisticated software as well as technical expertise in freehand scripting (9, 10, 12, 13). Simmons et al. estimated the percent of functional volume preservation by a cylindrical volume ratio method (7). It only took approximately 5 minutes for each patient but was limited for kidneys with substantial irregular defects. Chan et al. indicated that intraoperative visual assessment of functioning residual renal parenchyma by experienced surgeons who possess an educated cognition was the most accurate predictor of postoperative renal function (11). Tobert et al. compared the accuracy of surgeon assessment of volume preservation to those of 3-dimensional imaging and cylindrical model-based functional volume preservation in predicting postoperative renal function (18). They found that surgeon assessment of volume preservation was more efficient with accuracy comparable to those of more time intensive alternatives. Nevertheless, visual assessment is subjective in nature and there may be variance among different surgeons.

Shin et al. raised the idea of using a mathematical model to calculate the RAIV and found that RAIV had a good correlation with the absolute and percent change in eGFR (14). For the purpose of better predicting functional outcome, we take into consideration the functional renal volume and propose a new formula to calculate PRAIV. Assuming the tumor as a sphere and bilateral kidneys as symmetrical ellipsoids, we calculate PRAIV using integral calculus. Six parameters, namely, tumor radius, depth of invasion, width of resection/ischemization, height, width, and length of kidney, were required in our formula. According to our study, though both RAIV (p=0.04) and PRAIV (p<0.001) were correlated with percent reduction of eGFR on univariate analysis, RAIV (p=0.5) lost its correlation on multivariate analysis. In other words, lesser PRAIV means more percentage of renal parenchyma preserved with subsequently better postoperative renal function. PRAIV could serve as a more comprehensive and accurate predictor of renal functional change compared with RAIV. The influence of PRAIV on renal functional change was also greater than that of C-index, while RENAL and PADUA exerted only marginal influence (Table-2). In addition, we found that age, a potential determinant of nephron quality, was correlated with percent reduction of eGFR, though the significance was less than PRAIV (R^2^=0.073 and 0.194, respectively).

Yossepowitch reported that cold ischemia time was associated with early postoperative eGFR changes, but not with eGFR decrease 12 months after surgery (19). Lane et al. also stated that when percentage of parenchyma spared was incorporated into the analysis, duration of ischemia time, either cold or warm ischemia, lost significance in determining ultimate renal function (8). Other studies indicated that when warm ischemia was kept less than 20 to 25 minutes or hypothermia was used, ischemia injury had a less pronounced role in determining renal function (12, 17). In line with the literature, the mean cold ischemia time of our study was 40.6 minute, and it was not a significant predictor of percent reduction of eGFR in the long term.

A major limitation of our study was that the renal tumor model was built geometrically. In fact, the tumor and kidney could hardly be a true sphere and ellipsoid, and the volume of renal cysts and collecting system should be adjusted in estimating functional renal volume. Besides, the calculation process using integral calculus was a little complicated. Nevertheless, our geometric model and formula provided an intuitive concept in estimating PRAIV. More importantly, our results reemphasized the great influence of renal quantity on the functional outcome after PN.

Another challenge to our results is that we arbitrarily defined the width of resection and ischemization as 0.5 centimeter in our series. Frankly speaking, the resection margin may not be identical all around the tumor, and the width of each bite varies suture by suture. Therefore, our results may be biased by inconsistent values of the width of resection and ischemization. Notwithstanding, as long as a histologic tumor - free margin is achieved, it is sufficient to get local tumor control in PN (20). So every effort should be made to render the resection margin as minimal as possible, and intraoperative ultrasound may be used for carefully planning before tumor dissection (21). Besides, the extent of renorrhaphy after tumor excision should be reduced to limit the area of tissue injury.

Other limitations of our study include the small sample size, retrospective nature, and single surgical approach. More validated results could be established if laparoscopic or robot-assisted PN are enrolled. In addition, in our study renal function was accessed using MDRD2 equation. Ideally, for patients with bilateral kidneys split renal function should better be evaluated by renal scintigraphy with technetium-99m-mercaptoacetyltriglycine (22).

## CONCLUSIONS

PRAIV determined by a geometric model is a significant predictor of renal functional change after PN. Using PRAIV based mostly on radiographic parameters, we can make a preoperative estimation of percent eGFR reduction for better patient consultation and surgical planning. Additional studies are required to access the applicability of PRAIV in predicting renal functional change after PN of various surgical approaches and in various institutions.

## ARTICLE INFO

Int Braz J Urol. 2017; 43: 80-6
